# Putting the “Biology” Back into “Neurobiology”: The Strength of Diversity in Animal Model Systems for Neuroscience Research

**DOI:** 10.3389/fnsys.2016.00069

**Published:** 2016-08-22

**Authors:** Joyce Keifer, Cliff H. Summers

**Affiliations:** ^1^Neuroscience Group, Division of Basic Biomedical Sciences, Sanford School of Medicine, University of South DakotaVermillion, SD, USA; ^2^Department of Biology, University of South DakotaVermillion, SD, USA

**Keywords:** animal models, diversity, neuroscience, central nervous system, evolution, conservation

## Abstract

Current trends in neuroscience research have moved toward a reliance on rodent animal models to study most aspects of brain function. Such laboratory-reared animals are highly inbred, have been disengaged from their natural environments for generations and appear to be of limited predictive value for successful clinical outcomes. In this Perspective article, we argue that research on a rich diversity of animal model systems is fundamental to new discoveries in evolutionarily conserved core physiological and molecular mechanisms that are the foundation of human brain function. Analysis of neural circuits across phyla will reveal general computational solutions that form the basis for adaptive behavioral responses. Further, we stress that development of ethoexperimental approaches to improve our understanding of behavioral nuance will help to realign our research strategies with therapeutic goals and improve the translational validity of specific animal models. Finally, we suggest that neuroscience has a role in environmental conservation of habitat and fauna that will preserve and protect the ecological settings that drive species-specific behavioral adaptations. A rich biodiversity will enhance our understanding of human brain function and lead in unpredicted directions for development of therapeutic treatments for neurological disorders.

## Introduction

Successful adaptation of animal behavior to environmental demands is reflected in the structure and function of nervous systems. Over the years of neuroscience research, we have come to realize the diversity of adaptive neural solutions that organisms have evolved to overcome environmental challenges and survive. In the burgeoning days of neuroscience research in the 1960’s and 70’s, then called “neurobiology”, researchers used a variety of vertebrate and invertebrate model systems to study the function of neurons and neuronal circuits. The fact that different animal models had particular strengths for addressing specific questions about nervous system function was appreciated. For example, the squid giant axon was selected by Hodgkin and Huxley (1952) to study ionic mechanisms underlying the action potential. The now classic frog neuromuscular junction preparation was developed to understand mechanisms of synaptic transmission (Del Castillo and Katz, 1954). Legions of neuroscientists exploited the “simpler” nervous systems of invertebrates with their identified neurons to define the synaptic and integrative properties of neural circuits controlling motor behavior and learning (Marder, 2012). Studies of these less complex model systems provided fundamental insights into the function of the more derived nervous systems of mammals including the human brain. More recently, there has been substantial reliance of the use of rodent models (mouse, rat) to study all aspects of brain structure and function in modern neuroscience research. In today’s neuroscience environment there is a lack of appreciation of, or reluctance to accept, less popular or atypical animal models. In this Perspective article, we argue that neuroscience research utilizing a rich diversity of animal model systems rather than just a few established ones significantly enhances our ability to discover fundamental molecular and physiological principles essential for nervous system function. Elucidation of highly conserved neuronal mechanisms is widely applicable to understanding normal human brain function and greatly facilitates understanding of neurological dysfunction and disease states.

## Animal Model Systems in Neuroscience Research

**Figure 1:**
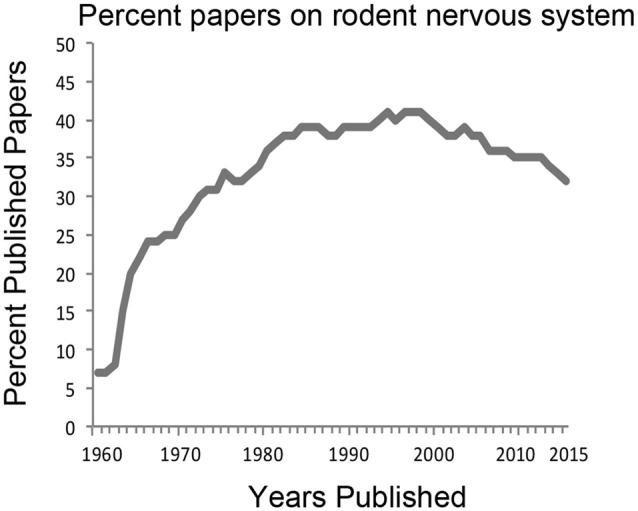
**History of the percentage of neuroscience articles published from work using rodent model systems.** PubMed search terms “rodent, nervous system” were used to derive the percentage of publications from the total number of articles published using “nervous system” between the years 1960–2015. For many decades, about 35–40% of all neuroscience studies have relied on rodent animal models.

Neuroscience research is currently dominated by studies of the rat and the mouse brain. This has been the trend for the last few decades. A search for “rodent, nervous system” from PubMed^1^ shows that approximately 35–40% of all research efforts are directed to those species (Figure 1). In 1970, 27% of published articles on the nervous system used rodent animal models and rose to 41% in the 1990s. In 2015, published work using rodents comprised 32% of all neuroscience articles. Interestingly, utilization of the mouse compared to that of the rat has surged in the last decade and a half due largely to development of powerful transgenic techniques. However, while laboratory mice and rats are housed with standard facility designs and care protocols reducing costs for the universities, they are also highly inbred and have been disengaged from natural environments for many generations thereby lacking genetic and behavioral diversity. Current research on other model systems such as *Aplysia*, *Drosophila*, zebrafish, reptiles and birds each comprise less than 1% of the total number of published articles. This reliance on rodent models for neuroscience research has led some to conclude that this trend severely limits the scope of our overall understanding of brain structure and function, especially in the context of evolution of the human brain (Preuss, 2000; Manger et al., 2008).

Understanding the function of the human brain and dysfunction in disease states is a primary goal of neuroscience research. However, we are restricted in the questions that can be asked when studying humans. Specific animals are selected for study because they are particularly suitable for addressing basic questions about brain function, but the research also has inherent value in conservation and management strategies for that species. While mice and rats are well characterized and bred for research, they are not always the best models for all areas of neuroscience investigation. For example, song learning in juvenile songbirds closely mimics human speech learning (Mello and Clayton, 2015). The neurobiology of birdsong has developed into a rich field of study that tells us much about auditory perception, learning and memory and cognition. More realistic hypotheses for understanding neural substrates underlying long-range spatial navigation in natural environments have also emerged from studies of foraging bats (Geva-Sagiv et al., 2015). An interesting result from this work is that the spatial two-dimensional coding representation observed in place cells and grid cells in the hippocampus in studies of rodents in small laboratory cages misses the more complex three-dimensional multiscale representation required for real-world navigation over long distances. Another important result from the same laboratory (Yartsev et al., 2011) also emphasizes species differences by showing that grid cells are formed in the entorhinal cortex of bats in the absence of theta oscillations, which are ubiquitous in rodent entorhinal cortex, and were previously believed to be required for the formation of grid cells. These results derived from bats argue against a major class of computational models of grid cells based on theta oscillations which may be the result of rodent whisking rhythms (Grion et al., 2016; Kleinfeld et al., 2016) rather than a universal property of hippocampal activity in all species. Finally, studies using the turtle have lead to elucidation of detailed cellular and molecular mechanisms underlying vertebrate associative learning (Ambigapathy et al., 2015; Keifer and Zheng, 2015). It is because of the remarkable ability of the turtle nervous system to withstand hypoxic conditions (since they are a diving species) that large portions of the central nervous system can be removed and studied in a dish for prolonged periods of time. This allows cellular analysis of the function of large portions of intact neural circuits as well as manipulation by pharmacological agents during a neural correlate of classical conditioning. Such *in vitro* experiments are not possible in mammalian species except in slices. While the neuroanatomical features of such animal models have undergone diversification, the physiological and behavioral species-specific specializations across the animal kingdom is what makes some animal models more attractive for specific areas of study than others.

## Recognition of Conserved Core Mechanisms for Gene Regulation

With the emergence of rapidly developing next generation sequencing technology and bioinformatics tools, numerous species (if not all) and particularly atypical model organisms for neuroscience research have become amenable to mechanistic investigations of gene function, expression and regulation in the brain. High throughput sequencing of genomes from numerous representative species is currently underway and data are now freely available in online databases such as GenBank. Emerging evidence indicates that tissue-specific core gene expression is highly conserved across vertebrates. When different tissues are compared across fish, frog, chicken, mouse and human (Chan et al., 2009), one-third of all expressed genes are found to be orthologous (similar genes found in different species, which while not identical, can be traced back to a common ancestral species). Importantly, molecular mechanisms underlying gene expression in brain share common features throughout animal species even though with increased size of genome complexity in mammals and human gene regulation mechanisms are correspondingly expanded. This is particularly true for the transcriptional machinery as shown by genome-wide data across phyla (Hahn, 2004; Adelman and Lis, 2012; Koster et al., 2015). Transcription factors are critical regulators of gene expression and many of these are highly conserved in structure and signaling function even though there is divergence in binding sites for specific transcription factors (Villar et al., 2014). The cAMP-response element-binding protein (CREB), for example, is an evolutionarily conserved transcription factor that is considered to be a master regulator of cellular responses to environmental stimuli as well as learning and memory (Lakhina et al., 2015). This regulator, like other so called “memory genes” (Harvey-Girard et al., 2012; Rittschof et al., 2014; Mello and Clayton, 2015), is essential for learning processes of organisms as diverse as *Aplysia*, worm, honey bee, bird, turtle, mouse and human. Learning-related epigenetic modifications in chromatin surrounding DNA are also shared across a variety of invertebrate and vertebrate model organisms (Levenson and Sweatt, 2006; Xiao et al., 2012; Ambigapathy et al., 2015). A recent study showed that even complex social behavioral responses to aggression during territory intrusion share common molecular mechanisms in terms of chromosome reorganization, transcription factor activation and neuroendocrine signaling across the honey bee, stickleback fish and mouse (Rittschof et al., 2014). Taken together, such findings have led to the emerging conclusion that core molecular components are major regulators of learning and memory, social behavior, and responses to stress that are evolutionarily conserved with expansion related primarily to species-specific behavioral adaptations. An understanding of basic core mechanisms will reduce the complexity of analysis of a bewildering array of proteins and regulators involved in specific processes, for example, transcription. Hence, future investigations of the molecular basis of specific behaviors among a diversity of species and model animal systems will give us a powerful means to recognize and detail the conserved core mechanisms that are fundamental to neuronal function and essential to human brain function.

## Deep Homology in Neural Networks

In addition to mechanisms underlying transcriptional regulation and gene expression, neural circuits are evolutionarily conserved in basic structural design but uniquely elaborated according to species-specific specializations (Preuss, 1995). An example is the common design of neural circuitry for motion detection in the fly and the mouse (Borst and Helmstaedter, 2015). The detection of motion in the visual scene is one of the earliest and most fundamental computational processing stages in vision. In both species, signals from the photoreceptors are divided into ON and OFF pathways and relayed to deeper levels in the CNS where cells become directionally sensitive. Once direction is established, this information is fused at the next synapse in both organisms to form a motion signal in one of the four primary directions. The organizational features and flow of information through different processing layers is schematically very similar in design between the fly and mouse. This is an indication of the robustness of this particular computational solution to the problem of motion detection and establishes the fly as a powerful model for studies of visual motion analysis. Another instructive example of common circuit design for a specific behavioral action is the remarkable homology of the arthropod central complex and vertebrate basal ganglia (Strausfeld and Hirth, 2013). Here, it has been proposed that the organization of the insect protocerebra forms excitatory and inhibitory pathways subject to modulation by dopaminergic inputs that are homologous to the mammalian basal ganglia in structure and in motor function. The authors concluded that adaptive motor program selection is a trait common across phyla and generated by homologous neural circuits. Many conserved features of brain organization are observed throughout amniotes. For example, topographic analysis of thalamocortical projections using tract tracing in turtles showed a medial to lateral specificity of connections (Zhu et al., 2005). This finding indicates the presence of a functionally segregated pattern of thalamocortical projections that is a conserved feature of brain organization among amniotes. Moreover, the avian brain shares similarities in the neural control of learning and memory (Mello and Clayton, 2015) as well as in features of cognition (Clayton and Emery, 2015) compared to that of humans. Studies reveal that songbird learning shows remarkable similarity to human speech learning. Across all vertebrates, behaviorally significant brain regions and connections are maintained over 450 million years of evolution (O’Connell and Hofmann, 2011, 2012). This extraordinary conservation of function yields common homologous neural circuits controlling fundamental processes such as reward and social decision-making. The result is that complex social processes are represented in neural pathways of non-mammalian vertebrates (Korzan and Summers, 2007; Oliveira, 2013). However, brain structures unique to individual species have also emerged. Such is the case made for the prefrontal cortex of primates (Preuss, 1995; Wise, 2008). While homologous regions in the frontal lobe are shared by rats and primates, only primates have evolved a “granular” prefrontal cortex that is the essence of the behavioral flexibility displayed by them. However, it is also true that behavioral flexibility is also displayed by animals that do not have a granular prefrontal cortex or even a neocortex, for example, turtles (Grisham and Powers, 1989) and fish (Carpenter and Summers, 2009). Diversity informs us by revealing common computational solutions for behavior that span species. Continued analysis of homologous neural networks controlling behavior as an analogy for human brain function will reveal general computational solutions (Striedter et al., 2014) that form the basis for adaptive behavioral responses.

## Toward Understanding Behavioral Nuance

Development of therapies and drugs for neural disorders has driven scientific methodologies increasingly toward rapid high-volume results and a reduction in the number and kind of animal models used. However, the methods by which we approach behavioral experiments should be carefully attuned to the ecological profile of the animal being examined (Blanchard et al., 2001, 2013; Robertson et al., 2015). An ethoexperimental approach, such as that which can be found in the Blanchards’ Visible Burrow System and BTBR mouse facial expression/nose-nose interaction tests for autistic behavior, as well as the Stress Alternatives Models from the Summers’ lab, captures the dynamism of a natural environment and mechanisms that are specific to highly nuanced behaviors (Pearson et al., 2016). The success of ecologically and ethologically relevant models for elucidating neuropsychiatric and neurological disorders underscores the need for further development of new models that incorporate similar dynamics (Blanchard and Blanchard, 1989; Carpenter and Summers, 2009; Nestler and Hyman, 2010; Beery and Kaufer, 2015; Robertson et al., 2015; Smith et al., 2016). Therefore, we suggest that this approach provides the potential to address diverse neurological and psychiatric disorders by making use of the distinctive natural behavioral repertoire of any species that has elements of behavior and physiology that are driven by neural circuits involved in human disorders. The critical point here is not to simply try to match behavioral face validity from animal model to human translation, but to use natural behaviors belonging to the animal model and match neuroregulatory systems to the human condition (Young et al., 2012; Blanchard et al., 2013). As we continue to investigate the neural mechanisms involved in producing clinically relevant disorders, how we design model systems and behavioral paradigms, specifically by using an ethoexperimental approach to explore translational interactions of physiology, genetics, and environment on behavioral adaptations, will continue to inspire more realistic experimental approaches.

Complexity is always far more prevalent in human and animal behavior than we initially imagine. Derived from genetic background and evolutionary history, environmental stimuli, gene-environment interactions, and internal physiology, complexity is necessary to establish adaptive significance for the individual behavior (Tinbergen, 1963). Recent trends indicate that the number of animal models considered for behavioral studies are extremely limited and are paired with experimental protocols that depend on extremely simplified stimuli (painful or novel) and outcomes to produce high throughput designs for assessing translational relationships with human disorders, despite the lack of obvious complexity in task design (Blanchard and Blanchard, 1989). Examination of recent studies suggests they are of limited clinical validity (Nestler and Hyman, 2010; Haller and Alicki, 2012; Haller and Freund, 2013; Haller et al., 2013). Context and small discrepancies in behavior (nuance) often make distinctive differences in neural activity and the adaptive value of the response for the individual (Blanchard and Blanchard, 1969a,b; Blanchard et al., 1995, 2001, 2011, 2013; Defensor et al., 2011; 2012). We agree with an early statement by Lorenz (1958), that to understand the underlying mechanisms involved, the full range of behavior in contextually appropriate naturalistic settings is necessary. A pertinent example stems from an area highly investigated, sexual behavior in rats, typically using spare, but adequate, social and environmental conditions. However, when more natural and ethological conditions are applied, the results reveal additional highly nuanced behavior in both male and female rats, resulting in differential female receptivity as well as female and male fertility, in which the social and physical environment provides significant information about sexual interactions (Chu and Ågmo, 2015a,b; Chu et al., 2015). Therefore, behavioral paradigms and experimental designs created to study the neural and molecular regulators of distinguishing behaviors should come from critical analyses of nuance (Robertson et al., 2015). Parsing this continuum of behavioral nuance is no small task, as the experimenter must tie the complex and poorly understood symptomatology in humans to observable behaviors in animals. The key is to examine *natural* behaviors, using extant species in or related to their natural environments, where evolutionary adaptions make the behavioral outcome relevant to the animal and only secondarily related to the human malady under consideration (Blanchard et al., 2013). After relating animal behavior to human symptoms (face validity), effective models ought to also make use of therapies producing parallel results in animals and people (predictive validity), and relate the symptoms and treatments to the specific neurocircuitry and physiology involved (construct validity). For depression and anxiety, one of the hardest standards to meet is construct validity because the complex biological and behavioral substrates are only poorly understood. Often incongruous results stem from studies which seek to explain a single aspect of broadly multifaceted phenomena. Contemporary trials in clinical populations suggest that the translative power of most single niche tests for psychological disorders in animal models is low (Haller and Alicki, 2012; Haller and Freund, 2013; Haller et al., 2013). An innovative emphasis aimed at where the diversity of behavioral nuance between disorders and models converge may become the crucial element in assessing animal model validity (Blanchard et al., 2013).

## Alignment of Research Strategies with Therapeutic Goals

Recent meta-analyses of standard animal models for human psychiatric disorders provide clear evidence that customary laboratory-reared rodents do not produce outcomes that consistently translate well to effective clinical trials, much less to successful therapies (Holsboer and Ising, 2010; Haller and Alicki, 2012; Blanchard et al., 2013; Bukalo et al., 2014). These analyses have played a fundamental role in the realignment of research strategies by national funding agencies toward even more anthropocentric approaches and policies. Basic research and serendipity have always played critical roles in discovering new therapeutic approaches and tools. Alternative animal models have long been a part of the mix of basic research approaches that have yielded directed and serendipitous advances. Recent evidence suggests that classification of psychoses into distinctive groups based on biological markers have a better diagnostic capacity than the symptoms presented by patients (Clementz et al., 2016). The biological markers that were meant to produce a more unbiased classification of the psychoses have been developed, at least in part, using non-rodent or non-human animal models (Lindsley et al., 1950; Garcia-Austt, 1954; Gusel’nikov, 1956; Fite et al., 1979). The most important idea is that the more biological markers that can be developed, the more efficient our diagnostic capacity may become for a variety of maladies including psychological disorders. The greatest opportunity for discovering the necessary molecular or neurochemical markers is to include the greatest variety of animals in our research. In a rational approach to increase biological markers through animal models, consider that most rodent models are based on highly inbred rats and mice. These inbred strains do not reflect the full range of behavioral responses of natural populations and therefore almost certainly do not demonstrate the full range of physiological or molecular responses of wild animals (Sgoifo et al., 1996; de Boer et al., 2003; Coppens et al., 2014). It is likely that one reason that models derived from laboratory-raised animals are not well translated into clinical successes is that the human condition is far closer to that of wild animals than to those of inbred rodents that are bred, raised and held under conditions that can only be described as highly deprived with respect to sensory input and behavioral opportunities (Van de Weerd et al., 1997; Kavelaars et al., 1999; van den Berg et al., 1999; de Boer et al., 2003; Meeusen, 2005; Korte et al., 2007; Balcombe, 2010; Blanchard, 2010; MacGillivray et al., 2012; König et al., 2015; Smith et al., 2016).

## A Broader Appreciation of Psychiatric Disorders

About three decades ago, significant work in non-mammalian models began to link social stress, neural and endocrine systems with acute and chronic emotional behavior (Greenberg et al., 1979, 1984a,b; Greenberg and Crews, 1990). The work led to evidence that linked specific stress-induced monoamine neurochemistry and hormonal changes to behavioral phenotypes and outcomes associated with social rank relationships (Summers and Greenberg, 1994, 1995; Summers et al., 1997). What followed were numerous studies that demonstrated similar changes in non-mammalian nervous and endocrine systems following social defeat paradigms, as are seen in human cases of depression (Summers et al., 1998, 2003a). It also became clear that behavioral phenotypes centered on stress coping strategies were highly conserved across vertebrate taxa, including humans, and they represented a clear mechanistic link between behavioral responses and affective states (Koolhaas et al., 1997, 1999, 2007; Pottinger and Carrick, 1999; Øverli et al., 2004a,b, 2007). A seminal *Society for Integrative and Comparative Biology* symposium in 2001 analyzed standard and comparative animal models to ask the question: *Is Stress More than a Disease?* (Carr and Summers, 2002). Several important conceptual advances were revealed suggesting directions for the focus of future stress-related study including psychological disorders: (1) Examining the comparative endocrinology and evolution of stress helps to identify common themes of neuroendocrine integration and control; (2) Comparing widely used mammalian models with other vertebrates in laboratory or field settings facilitates recognition of adaptations of behavior, reproductive strategy, and life history; (3) Non-mammalian species are sometimes better models for unraveling complex neuroendocrine control mechanisms; and (4) Examining natural populations of animals provides insight into adaptive features of stress not previously observed when studying laboratory mammals. It is commonly accepted now that chronic stress is an etiological precursor to anxiety and depression, but prior to the development of the Social Defeat model of depression, results from several studies on lizards and fish made it clear that human affective behavioral inhibition occurring concomitantly with anxiety and depression is not unique (Øverli et al., 2004a,b, 2007; Summers et al., 2005a; Korzan et al., 2006; Korzan and Summers, 2007). Even more important is that the neurochemical, neuroendocrine and molecular changes associated with human depression and anxiety occur in the same brain regions of fish and lizards following social subordination stress (Summers and Greenberg, 1994; Summers et al., 1997, 1998, 2003a,b, 2005a,b,c; Summers, 2001; Summers and Winberg, 2006; Korzan and Summers, 2007; Robertson et al., 2015). Similarly, recent work on Rainbow trout and Atlantic salmon suggests that for individuals within a population, a suite of symptoms occur that are virtually identical to those of depressed human patients. This depressive syndrome is characterized by chronic brain serotonin (5-HT) dysfunction, increased cortisol production and behavioral inhibition (Riise et al., 2015; Vindas et al., 2016). This depressive state was demonstrated to facilitate tolerating risky social environments. Development of the Social Defeat model of depression in rodents followed the work in lizards and mirror the results seen in fish in which individuals susceptible to chronic social stress display depressive behavioral inhibition in a number of classical tests of anxiety and depression whereas resilient individuals do not (Berton et al., 2007; Krishnan et al., 2007; Lutter et al., 2008). The results from these experiments suggest that depressive and anxious affective states evolved long ago to minimize unpredictable stress exposure in vulnerable individuals and have been conserved across vertebrate phyla. Taken together, the comprehensive scope of results from widely varying vertebrate systems suggests that it is no longer appropriate to reserve the descriptive terms “depression” and “anxiety” for human patients alone. What is more, it is apparent that an understanding of the behavioral inhibition seen in comparative animal models, in combination with the important interpretation of reduced behavioral responses being ecologically and ethologically adaptive, may help advance our understanding of these previously considered human-only psychiatric disorders.

## Neuroscience has a Role in Environmental Conservation

Animals respond to their environments through sensory and motor systems combined with integrative neuroplasticity, all uniquely evolved to produce adaptive behavioral outcomes. Environmental conservation of specific habitats and niches preserves and protects the specific behavioral adaptations and underlying neural substrates of the fauna endemic to those habitats. Therefore, one of the great strengths of comparative neuroscience is that evolutionarily conserved neural systems are linked fundamentally to adaptive behavioral responses in identified ecological and ethological settings. As each animal is uniquely suited to a specific environment, there is much to be learned from analyzing the neural function of wild animals while taking into consideration the environments that provided the evolutionary grist to result in the specialized nervous systems unique to each species (Manger, 2011). As humanity has benefited greatly from the discovery of natural compounds effective in treatments for diseases such as cancer, a rich biodiversity in species and habitat is essential in understanding brain function and in developing novel therapeutic advances for neurological disorders.

## Author Contributions

JK and CHS wrote the article.

## Funding

CHS was supported by National Institutes of Health (NIH) grant MH104485 for his work on mice. This publication was supported by the National Institute of Mental Health of the National Institutes of Health under Award Number R15MH104485. The content is solely the responsibility of the authors and does not necessarily represent the official views of the National Institutes of Health or the United States Government.

## Conflict of Interest Statement

The authors declare that the research was conducted in the absence of any commercial or financial relationships that could be construed as a potential conflict of interest.

## Footnotes

^1^http://www.ncbi.nlm.nih.gov
